# Heat or Cold: Which One Exerts Greater Deleterious Effects on Health in a Basin Climate City? Impact of Ambient Temperature on Mortality in Chengdu, China

**DOI:** 10.3390/ijerph13121225

**Published:** 2016-12-10

**Authors:** Yan Cui, Fei Yin, Ying Deng, Ernest Volinn, Fei Chen, Kui Ji, Jing Zeng, Xing Zhao, Xiaosong Li

**Affiliations:** 1 Department of Epidemiology and Health Statistics, West China School of Public Health, Sichuan University, Chengdu 610041, Sichuan, China; ian_cui@foxmail.com (Y.C.); westsilverhx@163.com (F.Y.); jacky2183@163.com (F.C.); 2 Sichuan Centre for Disease Control and Prevention, Chengdu 610041, Sichuan, China; denghjs@aliyun.com (Y.D.); cdjk@163.com (K.J.); sccdc_zj@163.com (J.Z.); 3 Department of Anesthesiology, Pain Research Center, University of Utah, Salt Lake City, UT 84108, USA; ernest.volinn@hsc.utah.edu

**Keywords:** cold, heat, impact, mortality, comparison, basin

## Abstract

*Background*: Although studies from many countries have estimated the impact of ambient temperature on mortality, few have compared the relative impacts of heat and cold on health, especially in basin climate cities. We aimed to quantify the impact of ambient temperature on mortality, and to compare the contributions of heat and cold in a large basin climate city, i.e., Chengdu (Sichuan Province, China); *Methods*: We estimated the temperature-mortality association with a distributed lag non-linear model (DLNM) with a maximum lag-time of 21 days while controlling for long time trends and day of week. We calculated the mortality risk attributable to heat and cold, which were defined as temperatures above and below an “optimum temperature” that corresponded to the point of minimum mortality. In addition, we explored effects of individual characteristics; *Results*: The analysis provides estimates of the overall mortality burden attributable to temperature, and then computes the components attributable to heat and cold. Overall, the total fraction of deaths caused by both heat and cold was 10.93% (95%CI: 7.99%–13.65%). Taken separately, cold was responsible for most of the burden (estimate 9.96%, 95%CI: 6.90%–12.81%), while the fraction attributable to heat was relatively small (estimate 0.97%, 95%CI: 0.46%–2.35%). The attributable risk (AR) of respiratory diseases was higher (19.69%, 95%CI: 14.45%–24.24%) than that of cardiovascular diseases (11.40%, 95%CI: 6.29%–16.01%); *Conclusions*: In Chengdu, temperature was responsible for a substantial fraction of deaths, with cold responsible for a higher proportion of deaths than heat. Respiratory diseases exert a larger effect on death than other diseases especially on cold days. There is potential to reduce respiratory-associated mortality especially among the aged population in basin climate cities when the temperature deviates beneath the optimum. The result may help to comprehensively assess the impact of ambient temperature in basin cities, and further facilitate an appropriate estimate of the health consequences of various climate-change scenarios.

## 1. Introduction

The association between ambient temperature and mortality has been demonstrated in many parts of the world [1]. Due to global climate change, the characteristics of extreme weather events have changed substantially during the past several decades, with an increase in the frequency, duration, and intensity of heat waves but a decrease in the frequency, duration, and intensity of cold waves [2,3]. Given this trend, there is much concern about the health impact of heat. Many previous studies have investigated the associations between heat and mortality in the United States, Europe, and China. For example, a study of 15 European cities found that an increase of 3.12% in the Mediterranean region and 1.84% in the north-continental region in all-cause mortality was associated with a 1 °C increase in the maximum temperature above the city-specific thresholds [4]. Heat-related mortality, however, may be contingent upon designated characteristics. For example, some studies have found that the cities with lower annual mean temperatures have greater heat-related mortality [5,6]. Such a finding suggests geographic variability in excess mortality attributable to high temperature [7]. For the most part, however, studies on the relationship between climate and mortality have been conducted in affluent western countries, and the question is whether findings from them extend to China, the most populous country in the world. The recent literature suggests that such findings often are not applicable to China [8].

Concerning the effect of extremes in whether events on mortality, this relationship has also been examined in a large number of studies [9]. Consistent with the upsurge in scholarly and media attention, they mainly have focused on heat waves to demonstrate the effect of global warming [10]. Fewer studies have examined the health effect of cold spells [11], and, accordingly, cold has drawn less attention than heat as a health hazard [12]. A study on the global association of cold spells and adverse health effects, however, has indicated that cold spells are associated with increased mortality from all or all non-accidental causes, cardiovascular diseases and respiratory diseases [13]. Another study reported that there is an association between cold temperatures and mortality from cancer, which is most marked among the elderly [14]. Fewer studies have been conducted on the effects of cold than the effects of heat, but those studies that have been conducted indicate that cold exerts the greater effect on mortality [15]. Recent epidemiological studies show that mortality caused by cold temperature is comparable to that caused by the severest heat temperature [16]. Although a few studies conducted in China reported the effect of the 2008 cold spell on health [17], the effects of cold in China afterward constitutes an issue that, in relation to its possible impact on the population, remains under-investigated.

Existing studies have provided estimates of mortality attributable to either heat or cold, but a question that remains largely unexplored is the simultaneous assessment of the effects of both heat and cold and the relative effects of each one on human health, especially in basin climate cities. Previous studies found that some people susceptible to heat effects are those with advanced forms of illness who may be expected to die anyway within a short period [18,19], while cold effects were mostly positively sustained and more evenly distributed across the several weeks [18]. The relationship between temperature and mortality has been viewed as U-shaped [20], with increased risks for extreme heat and cold temperatures. When heat or cold temperature last for a few days or more there may be additional risks because of the extra impact on body’s systems [1]. In view of the evidence that extremes in both cold and heat affect health, the issue becomes more generally to characterize with some exactitude the relationship between temperature and mortality.

Chengdu, as the largest city with a very large population in Southwest China, may greatly benefit from a comprehensive understanding of health effect of temperatures. Chengdu is located in the Sichuan basin, and the overall topography of the region is complicated, with particular and various microclimates. There are sharp downslope winds from the mountain summits to the basin beneath, with vortex type wind fields in the middle of basin, and the overall pattern produces an accumulation of moisture in the basin [21]. On cloudy days, atmospheric stratification of the Sichuan basin mainly is neutral, with an annual average ratio of about 60% and little change with the seasons. There is primarily radiation inversion in each season in the basin. The temperature inversion frequency is highest in winter (above 40%) [22,23]. This may lead a different impact of ambient temperature on health. New evidence is needed to implement accurate interventions in vulnerable regions and populations.

Our aim in this paper was thus to investigate the contributions of ambient temperature, specifically cold as well as heat, on mortality and to sort out the effects of each specifically in a large basin area of China. The result may help to comprehensively assess the impact of ambient temperature in the basin city, and further facilitate an appropriate estimate of the health consequences of various climate-change scenarios.

## 2. Materials and Methods

**Figure 1:**
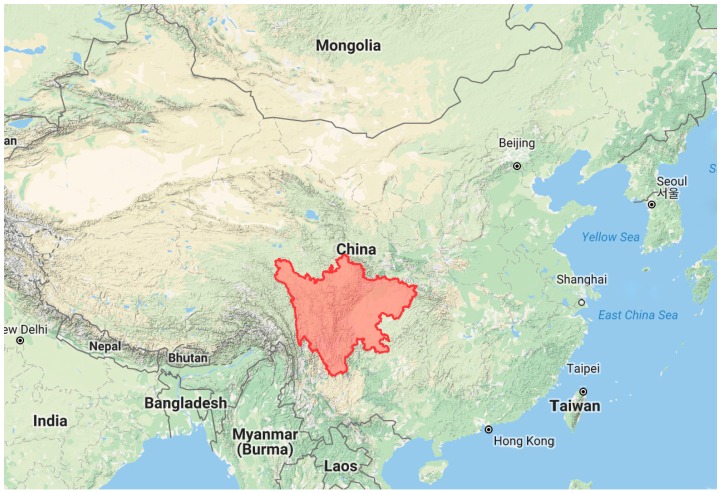
Partial map of China (the area in red is Sichuan Province, which located in southwest China).

**Figure 2:**
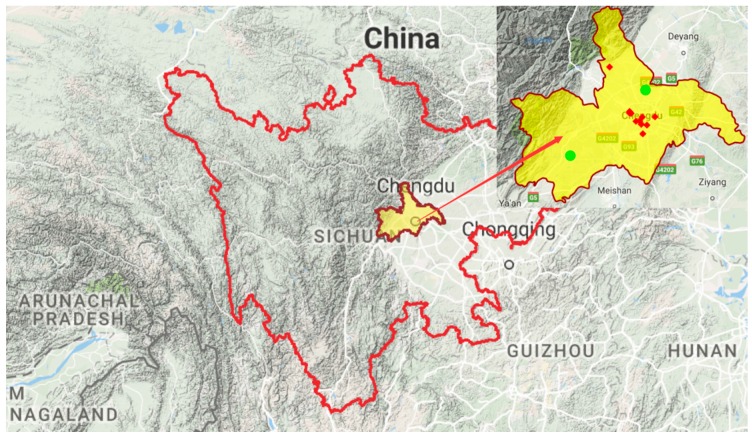
Terrain map of Sichuan Province (the area in yellow is Chengdu, our study area. Red points indicate the air pollution monitoring stations and green points are the meteorological observation stations).

The study population comprised all residents in Chengdu, which is in a basin area and, with a population of 14.65 million, is the largest city in the southwestern province of Sichuan (see Figure 1 and Figure 2). The surface area of Chengdu is 14,605 square kilometers. The study region consisted of ten districts, four county-level cities and five counties.

### 2.1. Data

We obtained daily records of deaths for Chengdu City in China between 1 January 2011 and 31 December 2014. The death surveillance stations from Sichuan Center for Disease Control and Prevention provide coverage of all 19 districts. Death records indicated the date of death, the cause of death coded by the International Classification of Diseases 10th (ICD10) revision. Among the 276,024 non-accidental deaths (NAD) in Chengdu (2011–2014), 85,087 (30.8%) died of cardiovascular diseases and 70,960 (25.7%) died of respiratory diseases.

The two meteorological monitoring stations in the suburban areas belong to China Meteorological Data Sharing Service System and provided daily meteorological data, including mean temperature, relative humidity, pressure, and wind speed for this project. 

To adjust potential confounding effects of air pollutants, we obtained daily air pollution data for 2011–2014 from the 12 monitoring stations of the Sichuan Environment Monitoring Center. The air pollution data included 24-h average values of ambient PM10, NO_2_, and SO_2_. The complete daily average value alignment was obtained through imputation of missing data on the basis of linear interpolation.

### 2.2. Analysis of Temperature-Mortality Relationship

#### 2.2.1. DLNM

We estimated the relationship between daily mortality and ambient temperature. As daily mortality counts generally follow an overdispersed Poisson distribution, we used a distribution lag model with a quasi-Poisson regression to evaluate the health effect of heat and cold while adjusting for the temperature at different lag days. We controlled for long time trends and day of week. The model used the following formula:

(1) $$\mathrm{Ln}\left[E\left(Y_{t}|X\right)\right]=\alpha +cb\left(Temperature,\;lag\right)+ns\left(Time_{t},7\right)+DOW_{t}$$

where *t* refers to the day of the observation; $E\left(Y_{t}|X\right)$ denotes estimated daily NAD on day *t;* α is the intercept; ns() denotes the cubic smoothing spline; cb(Temperature, lag) is a matrix obtained by applying to temperature; *lag* refers to the maximum lag days; $Time_{t}$ is the day of calendar time on day *t,* with *7* degrees of freedom (DF) per year; $DOW_{t}$ is the day of the week on day *t.* The model was fitted using a quadratic spline with two equally spaced knots for temperature and a natural spline with an intercept and three internal knots placed at equally spaced values in the log scale. We extended the lag period to 21 days [24] to include the long delay of effects of cold and to exclude deaths that were advanced by only a few days (harvesting effect). The relationship between temperature and mortality was summarized as the exposure-response curve of relative risk (RR) accumulated across all lags. The Akaike Information Criterion for quasi-Poisson (Q-AIC) values was used for choice of the DF. We tested these modelling choices in sensitivity analysis.

#### 2.2.2. Attributable Risk from DLNMs

Attributable fraction (AF), which takes into account cold-risk but also the number of days on which that risk is observed, is the most useful indicator of cold-related health burdens. The number of deaths on each day of the series attributable to ambient temperature was computed, using as reference and cut-off the Minimum Mortality Temperature [24], which is the value of temperature at which mortality risk is the lowest. We used a backward perspective, assuming the risk at time *t* as attributable to a series of exposure events in the past [25].

We conducted a stratified analysis by sex and age and tested for statistically important differences between effect estimates of the strata of a potential effect modifier by calculating the 95% confidence interval as shown below:

(2) $$\left({\hat{Q}}_{1}-{\hat{Q}}_{2}\right)\pm 1.96\sqrt{{\hat{SE}}_{1}^{2}+{\hat{SE}}_{2}^{2}}$$

where ${\hat{Q}}_{1}$ and ${\hat{Q}}_{2}$ are the estimates for the two categories, and ${\hat{\mathrm{SE}}}_{1}$ and ${\hat{\mathrm{SE}}}_{2}$ are their respective stand errors. Regardless of significance, we considered modification of effect by a factor or two to be important and worthy of attention [26].

#### 2.2.3. Separating Attributable Components

The minimum mortality temperature (MMT), which corresponds to a minimum mortality percentile across the entire temperature spectrum, was derived from the prediction of the overall cumulative exposure-response association. We referred to this value as the optimum temperature, and deemed it the reference for calculating the attributable risk. For each day of series, we used the overall cumulative RR corresponding to each day′s temperature to calculate the attributable deaths and fraction of attributable deaths in the next 21 days, using the method described previously in Gasparrini’s study [25]. According to the range r_1_ = (temp_min_, temp_optimum_), we derived the attributable risk of cold. Also, the attributable risk of heat could be derived from the range r_2_ = (temp_optimum_, temp_max_). We further separated the temperature range into moderate and extreme contributions by defining extreme cold and heat as temperatures lower than the 2.5th location specific percentile (extreme cold) and higher than the 97.5th location-specific percentile (extreme heat). Temperatures from the 2.5th percentile to the MMT and temperatures from the MMT to the 97.5th percentile are defined as the moderate cold and heat. And then we calculated the attribution fractions of every component.

#### 2.2.4. Computing Uncertainty Intervals

An analytical formula for confidence intervals of attributable risk measures is not easily produced. Although approximated estimators have been proposed, the most straightforward approach is to rely on the interval estimation obtained empirically through Monte Carlo simulations [27]. We obtained 50,000 random samples from the assumed normal distribution, and interpreted the related 2.5th and 97.5th percentiles of such distributions as 95% confidence intervals (CI).

### 2.3. Sensitivity Analysis

A series of sensitivity analysis was performed to test the robustness of our results. We changed different degrees of freedom for each year and adjusted the main pollutants and meteorological data in the model. All analyses were conducted with R software (version 3.2.5; R Foundation for Statistical Computing, Vienna, Austria, 2016) using the package “dlnm”.

## 3. Results

Data of weather conditions, death counts and individual characteristics in Chengdu from 1 January 2011 to 31 December 2014 are summarized in Table 1.

**Figure 3:**
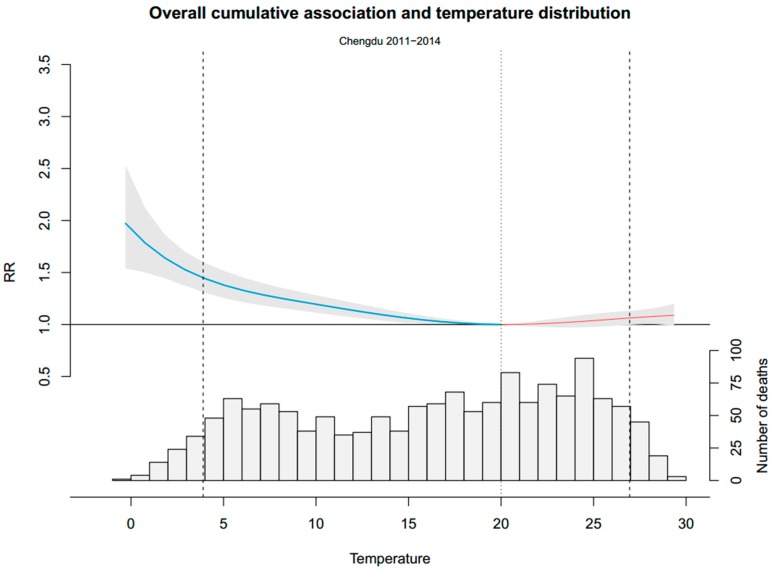
Overall cumulative exposure-response association in Chengdu City. The blue part of the curve is the exposure-response association (with 95% empirical confidence interval, shaded grey) of cold, and the red one presents the heat. The dotted line is minimum mortality temperature and the dashed lines are the 2.5th and 97.5th percentile. RR represents as the relative risk.

From 2011 to 2014, a total of 276,024 NAD were recorded. The two main causes of NAD, cardiovascular disease and respiratory disease, accounted for 85,087 and 70,960, respectively. On average, there were 188.9 daily NAD, 58.6% of which were male and 52.4% of which were older people. The average temperature was 16.2 °C, with ranges of from −0.3 °C to 29.4 °C. Figure 3 shows the overall distribution of temperatures and the cumulative association between temperature and mortality in our data. The overall cumulative association traced a fall J-shape curve. RR on cold days were relatively high, while RR were close to 1 on hot days. Moreover, most of the RR on hot days are not statistically significant. The temperature distributions show that the cold temperature range, although characterized by a high RR, consisted of only a small proportion of days. The minimum mortality percentile ranges were at about the 62nd percentile and the minimum mortality temperature was 20 °C. Risk increased slowly and linearly for cold temperatures between the minimum mortality temperature and the 5th percentile of the temperature. Below the 5th percentile of the temperature, which we called extreme cold days, risk increased exponentially.

Table 2 shows the estimated attribution fraction calculated as total and as separated components caused by heat and cold temperatures. Overall, the total fraction of deaths caused by both heat and cold was 10.93% (95%CI: 7.99%–13.65%). Taken separately, the effect of cold was significant and was responsible for most of the burden (estimate 9.96%, 95%CI: 6.90%–12.81%) and, in contrast, the effect of heat was small and non-significant (estimate 0.97%, 95%CI: 0.46%–2.35%).

**Figure 4:**
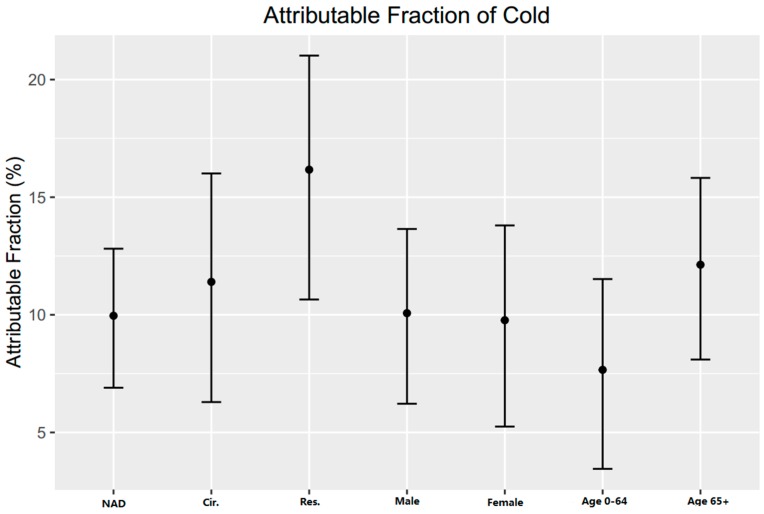
Attributable fraction of cold for different disease causes, sex and age group. NAD = non-accidental disease, cir. = cardiovascular disease, res. = respiratory disease.

Figure 4 shows the attributable fraction of cold in different groups. Respiratory diseases have a higher AF (19.69%, 95%CI: 14.45%–24.24%) than that of cardiovascular diseases (11.40%, 95%CI: 6.29%–16.01%). People aged above 65 have a higher AF (12.13%, 95%CI: 8.10%–15.82%) than that of people aged 0–64 (7.66%, 95%CI: 3.45%–11.52%). Male and female showed no significant difference (95%CI: −5.35, 5.96).

Changing the degrees of freedom for time trends yielded similar results. Also, we obtained similar results before and after adding the pollutants and meteorological data to the model (see Appendix A).

**Figure 5:**
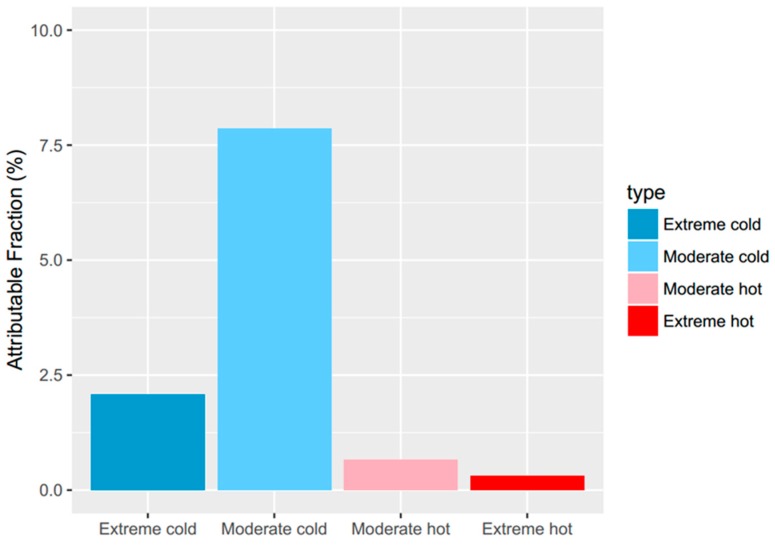
Fraction of non-accidental mortality attributable to moderate and extreme hot and cold temperature.

The attributable risk can be separated into components related to moderate and extreme temperatures (Figure 5). Extreme temperatures were responsible for a small fraction (2.39%, 95%CI: 0.35%–4.13%), while the moderate temperatures were responsible for most of the temperature-related deaths (8.54%, 95%CI: 5.87%–11.32%).

## 4. Discussion

Both high and low temperatures affect health in the large Chinese basin city of Chengdu. Although associations between ambient temperature and mortality have been documented in many countries [28], the comparison of heat and cold impact on human health remains limited, particularly in basin climate cities. To our knowledge, this is the first study comparing the mortality risk attributable to cold and heat temperature in China. Also, Chengdu is in a well-defined basin surrounded by mountains (Figure 2), which leads to the stalling of weather and its localization. It is a strategic for the study of effects of weather on health in basin cities.

Our findings show that far more of the mortality burden was caused by days colder than the optimum temperature (9.96%) compared with days warmer than the optimum temperature (0.98%). Furthermore, most deaths were caused by exposure to moderately hot and cold temperatures, which may result from the predominately high proportion of moderately hot and cold temperatures. This reflects other findings in the literature. Keatinge and Donaldson found that cold-related deaths are far more numerous than heat-related deaths in the United States, Europe, and almost all countries outside the tropics, and almost all of them are due to common illnesses that are aggravated by cold weather [29]. There is a mortality attributable to cold days which has been reported by many studies [13]; a finding in studies conducted in different places around the world [24] showed that temperature-attributable deaths caused by cold is 7.29% and by heat is 0.42%, i.e., twenty times higher. Similarly, a study conducted in Spain reported the effect of cold is five times greater than that of heat [30]. Though some families use the heating system in winter, the temperature difference between indoor and outdoor is large enough to threaten human health. Studies have evaluated mortality deviations from a base level as temperatures rose and fell by 0.1 °C increments in north Finland, south Finland, southwest Germany, The Netherlands, Greater London, north Italy, Athens and Greece, in people aged 65–74, finding that all regions showed more annual cold-related mortality than heat-related mortality [5]. Still another study found cold effects for the association of air temperature with cardiovascular and respiratory mortality. Regarding cold spell effects, a 5 °C decrease of the 15-day average temperature was associated with an RR of 1.036 (95% CI: 1.001–1.071) for cardiovascular mortality [31]. Evidence of this nature indicates that especially moderate low temperatures may impose more risk than high temperatures.

Figure 4 shows the attributable risk of cold. Why may cold exert a greater effect on respiratory mortality than cardiovascular mortality? Cold weather may serve to stimulate the respiratory system, which in turn may deplete and weaken the body’s resistance [32]. Other factors attributable to cold weather may be implicated as well. A previous study in Spain reported that, in contrast to heat, cold was indeed observed to have an effect on mortality associated with low temperatures in the youngest age groups [30]. However, we found a different result in our study area, that old people took more mortality burden in cold days. Especially when cold weather is extreme, the difference between indoor and outdoor temperatures becomes more pronounced, and people shut windows and thereby close off ventilation. Consistent with the sharp increase in mortality among the elderly in cold weather, thermoregulatory capacity decreases with age, and consequently increases the capacity of the upper respiratory tract to cool and mucosal membrane to dry [33]. In sensitive individuals, drying of the mucosa may lead to epithelial damage [34].

The correlation between cold conditions and smog is not as strong in Chengdu as cities further north. Some countries had highest effect estimates in moderate seasons, while others had highest effect estimates in cold seasons or hot seasons. While due to climate change, the balance of heat and cold related ill-health is likely to change over time [35]. On the one hand, the winter excess mortality is changing over time but, on the other, indoor heating can relieve the threat of cold in a certain degree. A recent study suggests that, although unstable weather is a continuous process, impacts on health may be better captured by considering the temperature variability when assessing the associations between temperature and human health [36]. Only time will reveal exactly how climate change-related shifts in temperature distributions will affect mortality trends.

Several limitations of this study should be mentioned. Firstly, we only focused on a single city. Further study using data from other basin cities is necessary to ascertain this study’s generalizability. Secondly, influenza and other morbidity conditions may fluctuate seasonally and independently of ambient temperature. Data on these conditions, however, were not available for this study. Thirdly, as with similar time-series studies, we used ambient temperature from meteorological stations to represent personal temperature exposure, which may lead exposure measurement errors. However, these errors are likely to be random and may result in an underestimation of the cold effect [37]. Fourthly, further research is needed to clarify how much of the excess mortality related to each component is preventable. Additionally, the estimated effects of temperature in the cold season can still be confounded by season if long lags are assumed [38].

## 5. Conclusions

We identified a substantial effect of ambient temperature on mortality in Chengdu. Cold was responsible for a higher proportion of deaths than heat, although a growing trend in public health research is to focus on heat waves, and, correspondingly, recent policies and interventions that have been aimed to reduce the impact of heat waves. We do not argue with this allocation of resources, but, rather, suggest that, in accordance with our results, they should be extended and readjusted to the whole range of temperature dependent effects, especially cold days in basin areas. The result may help to comprehensively assess the impact of ambient temperature in basin cities, and further facilitate an appropriate estimate of the health consequences of various climate-change scenarios.

## Figures and Tables

**Table 1:** Statistics of weather conditions and count of deaths in Chengdu, 2011–2014. NAD = non-accidental deaths.

Variables	Mean ± SD	Min	P_25_	P_50_	P_75_	Max
**Meteorological Factors**						
Temperature (°C)	16.2 ± 7.6	−0.3	9.3	17.3	22.8	29.4
Relative humidity (%)	74.8 ± 8.9	33.5	69.0	76.0	81.1	92.5
Pressure (hpa)	942.5 ± 7.2	925.2	936.4	942.7	948.2	963.8
Wind speed (m/s)	1.1 ± 0.4	0.2	0.8	1.0	1.3	3.0
**NAD**	188.9 ± 34.8	110.0	165.0	183.0	209.0	318.0
Cardiovascular	58.24 ± 13.7	21.0	49.0	56.0	67.0	118.0
Respiratory	48.6 ± 16.2	16.0	37.0	44.0	58.0	109.0
**Sex**						
Male	110.7 ± 21.1	61.0	96.0	107.0	123.0	191.0
Female	78.23 ± 16.8	36.0	66.0	76.0	88.0	150.0
**Age**						
Age (0–64)	89.9 ± 14.3	51.0	80.0	89.0	98.0	139.0
Age (65+)	99.0 ± 24.1	47.0	82.0	95.0	113.0	198.0

**Table 2:** Attributable mortality by disease causes, sex and age.

Group	Minimum Mortality Percentile	Total	Cold	Heat
NAD	62nd	10.93% (7.99%–13.65%)	9.96% (6.90%–12.81%)	0.97% (−0.45%–2.35%)
Cir.	72nd	12.09% (7.12%–16.48%)	11.40% (6.29%–16.01%)	0.69% (−1.69%–2.89%)
Res.	58th	19.69% (14.45%–24.24%)	16.17% (10.65%–21.02%)	3.53% (1.29%–5.59%)
Male	72nd	10.29% (6.56%–13.70%)	10.07% (6.22%–13.65%)	0.22% (−1.65%–1.99%)
Female	58th	11.78% (7.45%–15.69%)	9.77% (5.25%–13.80%)	2.02% (−0.01%–3.92%)
Age 0–64	100th	8.21% (4.14%–11.89%)	7.66% (3.45%–11.52%)	0.56% (−1.48%–2.47%)
Age 65+	68th	13.50% (9.69%–16.99%)	12.13% (8.10%–15.82%)	1.37% (−0.43%–3.09%)

Attributable mortality was computed as total and as separate components for heat and cold. The minimum mortality percentile, which corresponds to a minimum mortality temperature among the whole temperatures, was derived from the prediction of the overall cumulative exposure-response association. Age 0–64 group has a 100th minimum mortality percentile, because the tail of the curve towards to low which is different from others.

**Figure A1:**
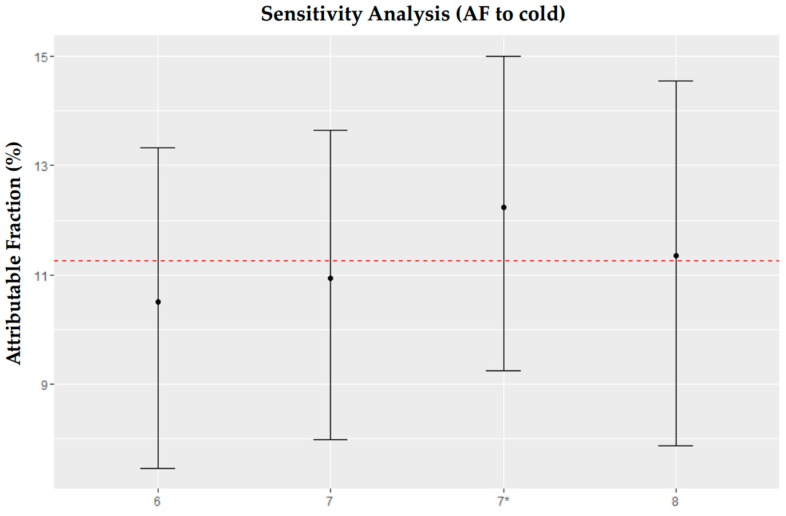
Attributable risk (AR) of cold for different degrees of freedom. * indicates pollutants and meteorological data were added to the model using a value of for the DF of time. The dashed line is the mean AR of the four groups.

## Appendix A

Data of air pollution from 1 January 2011 to 31 December 2014 in Chengdu is summarized in Table A1.

**Table A1:** Statistics of air pollution in Chengdu, 2011–2014.

Air Pollutant	Mean ± SD	Min	P_25_	P_50_	P_75_	Max
PM_10_	122.7 ± 76.3	16.0	72.0	104.0	150.0	862.0
SO_2_	28.4 ± 14.6	5.0	18.0	25.0	35.0	96.0
NO_2_	55.7 ± 19.2	15.0	42.0	52.0	66.0	144.0

To test the robustness of the models, we performed a sensitivity analyses by varying DF for time trends per year. Furthermore, pollutants and meteorological data were added to the model. Changing the degrees of freedom for time trends yielded similar results. Also, we obtained similar results before and after adding the pollutants and meteorological data to the model (Figure A1).
